# CircRNA AFF4 induced by KDM1A promotes osteogenic differentiation through FNDC5/Irisin pathway

**DOI:** 10.1186/s10020-022-00557-7

**Published:** 2022-11-18

**Authors:** Ansong Liu, Yong Chen, Da Zhong, Chenggong Wang, Mi Yu, Chao Liu, Zhijun Yang, Wenkang Chen, Ke Yin

**Affiliations:** 1 The First Affiliated Hospital, Department of Orthopedics, Hengyang Medical School, University of South China, No. 69 Chuanshan Road, Hengyang, Hunan 421001 China; 2 The First Affiliated Hospital, Department of Spine, Hengyang Medical School, University of South China, No. 69 Chuanshan Road, Hengyang, Hunan 421001 China; 3 Department of Orthopedics, Xiangya Hospital, Central South University, No.87 Xiangya Road, 410008 Changsha, Hunan China; 4 Hengyang Medical School, University of South China, Hengyang, Hunan 421001, China

**Keywords:** Circular RNA AFF4, KDM1A, Osteogenic differentiation, FNDC5, Irisin

## Abstract

**Background:**

Circular RNA (circ) AFF4 was documented to regulate osteogenesis but the underlying mechanism remains to be elucidated. The preliminary study showed that circ_AFF4 may promote osteogenesis via FNDC5/Irisin. Furthermore, the online prediction tool indicated the interaction of circ_AFF4, insulin-like growth factor-2 mRNA-binding protein 3 (IGF2BP3), FNDC5 and lysine (K)-specific demethylase 1 A (KDM1A). Therefore, this study aims to elucidate the relationships of KDM1A, circ_AFF4, IGF2BP3 and FNDC5/Irisin during osteogenesis.

**Methods:**

The alkaline phosphatase (ALP) activities and osteogenic-related factors were determined using ALP and alizarin red S (ARS) staining, real-time quantitative PCR(RT-qPCR) and western blot. Immunoprecipitation (RIP), pull-down assay and fluorescence in situ hybridization (FISH) were used to examine the interactions among circ_AFF4/IGF2BP3/FNDC5. A mouse *in vivo* model was utilized to further confirm the regulatory effect on bone formation.

**Results:**

Circ_AFF4 and KDM1A expression levels were increased during osteoinduction of BM-MSCs. Knockdown of circ_AFF4 and KDM1A significantly suppressed BM-MSC osteogenesis. We also proved that KDM1A directly bound to circ_AFF4 and FNDC5 promoter and induced circ_AFF4 and FNDC5 expression. Furthermore, circ_AFF4 enhanced the stability of FNDC5 by generating a circ_AFF4, IGF2BP3 and FNDC5 RNA-protein complex, and thereby induced Irisin and osteogenesis. The *in vitro* data was confirmed with *in vivo* model.

**Conclusion:**

These findings elucidate that KDM1A induces circ_AFF4, which promotes promote osteogenesis via IGF2BP3. This study indicates that circ_AFF4 may potentially represent a critical therapeutic target for the diseases.

**Supplementary Information:**

The online version contains supplementary material available at 10.1186/s10020-022-00557-7.

## Introduction

Irisin, a myokine derived from the cleavage of fibronectin III domain-containing protein 5 (FNDC5), is released into the bloodstream in response to muscle contraction (SousaA.L.D. et al. 2021). FNDC5, a transmembrane protein of skeletal muscle, is regulated by peroxisome proliferator-activated receptor γ coactivator 1α (PGC-1α) during exercise-related activities and release of irisin (Maak et al. 2021; Yang et al. 2021; Lourenco et al. 2019). This process activates mitochondrial energy regulation, fatty acid oxidation, fat browning, glucose metabolism and osteogenesis (Deng et al. 2020; Cho et al. 2021). Therefore, irisin, FNDC5 and PGC-1α are therapeutic targets of osteogenesis-related disease (Samy et al. 2015; Lin et al. 2021).

Circular RNAs (circRNAs), which is a class of small noncoding RNAs, form a covalently closed loop (Meng et al. 2017). Recently, with the improvement of RNA sequencing and computational technologies, many circRNAs were identified (Zhou et al. 2020). After that, circRNAs were demonstrated in publications to play functional roles in fundamental processes and may act as potential molecular makers of several human diseases, such as cancer (Meng et al. 2017). In our preliminary study, we found that circulation (circ) RNA AFF4 may promote osteogenesis via FNDC5/Irisin. Similar data were reported in a recent study. Liu et al. (2021). Using the computational prediction tool (Reif and Zacharias 2022), we also found that circ_AFF4 may potentially bind to insulin-like growth factor-2 mRNA-binding protein 3 (IGF2BP3). IGF2BP3, which belongs to a conserved IGF2 mRNA-binding protein family, was highly expressed in several tumor types including lung cancer, colon cancer, melanoma and cell carcinoma (Xueqing et al. 2020; Huang et al. 2021; Zhou et al. 2017). The abnormal upregulation of IGF2BP3 in tumorigenesis makes it a promising biomarker of tumor diagnosis (Lederer et al. 2014). However, the role of IGF2BP3 in osteogenesis remains to be unclear and has sorely been reported. Using online prediction tool (https://starbase.sysu.edu.cn/index.php), we identified that IGF2BP3 may bind to FNDC5. Considering the critical role of FNDC5 in circ_AFF4-mediated osteogenesis, we speculated that IGF2BP3 is potentially also involved in osteogenesis.

Histone modification is crucial in controlling the proliferation, migration and differentiation of MSCs (Ren et al. 2020). Lysine (K)-specific demethylase 1 A (KDM1A, also refers to as LSD1) is a regulatory protein of methylation, which is one type of histone modification (Kim et al. 2021). A previous publication reported that KDM1A used flavin adenine dinucleotide/dependent mechanism to demethylate H3K9me3 and regulated genes in MSCs (Rummukainen et al. 2022). KDM1A was also shown to have a complex role in different stages of osteogenic differentiation (Wang et al. 2018; Lv et al. 2016). It was proved that downregulation of KDM1A enhanced osteogenic differentiation both *in vivo* and *in vitro* (Wang et al. 2018; Sun et al. 2018; Ge et al. 2014). However, depletion of KDM1A reduced ALP activity and mineralization as shown by Wang et al. (2018). In addition, KDM1A was also predicted to bind to the promoter of circ_AFF4 and FNDC5 (http://bioinfo.life.hust.edu.cn/AnimalTFDB/#!/). The combination regulatory effects of KDM1A, circ_AFF4 and IGF2BP3 remain to be further elucidated.

Based on this data, we hypothesize that KDM1A binds to the promoter of circ_AFF4 and FNDC5 and mediates their expression. In addition, circ_AFF4 promotes osteogenic differentiation via IGF2BP3/FNDC5/Irisin axis.

## Materials and methods

### BM-MSC culture and characterization

Human bone marrow mesenchymal stem cells (BM-MSCs) were obtained from Procell (Wuhan, China) and cultured in Dulbecco´s Modified Eagle´s Medium (DMEM, Gibco, Grand Island, NY, USA) with 10% fetal bovine serum (FBS, Gibco) and 100 U/mL penicillin (Sigma, St Louis, MO, USA). Only BM-MSCs from passages 3–5 were used for this study.

Flow cytometry was used to characterize BM-MSCs using antibodies against CD45, CD14, CD90, CD73, HLA-DR and CD105 (BD, Biosciences, San Jose, CA, USA).

### Cell transfection

Based on previous studies, cell transfections were performed (Maak et al. 2021). Briefly, BM-MSCs were seeded 24 h prior to transfection. sh-IGF2BP3-1-4#, sh-KDM1A-1-4#, sh-circ_AFF4-1-4# or control shRNA were obtained from Genesee Biotech and were inserted into GV102 vectors and constructed by Genepharma (Shanghai, China). These vectors were transfected into BM-MSCs cells (5 × 10^6^ cells per well) using Lipofectamine 2000 (Invitrogen, Invitrogen, CA, USA) in 20 µL lipofectamine. After 6 h, the culture medium was changed to the normal medium. After 48 h, cells were used for the *in vitro* experiments. For lentivirus infection, lentiviruses carrying sh-KDM1A, circ_AFF4 overexpression lentiviruses, FNDC5 overexpression lentiviruses and correspondence control vectors were obtained from Genepharma and were used to infect BM-MSCs. Cells were selected with 2.5 µg/mL puromycin after 2 days post-transduction. 4–5 days later, puromycin was removed and cells were continuously cultured in the normal medium until fully recovered for animal studies.

### Osteogenic differentiation induction

Osteogenic Differentiation Medium BulletKit (Lonza, Basel, Switzerland) was used to induce BM-MSC osteogenic differentiation followed by manufacturer instructions. In brief, 1 × 10^4^ cells/well of BM-MSCs were seeded onto a 24-well plate until reach an 80% confluence. After complete removal of the medium, 1 mL/well of osteogenic medium (OM) was added. Cells cultured in normal DMEM were utilized as controls. The medium was changed every 3 days until 14 days. The cells were harvested for further analyses.

#### *In vivo* bone formation study

Adult C57BL/6J male mice that were between 12 and 14 weeks and weighing 25–30 g were given by Hunan SJA laboratory animal Co., Ltd (Changsha, China) and were kept in an animal facility (temperature 22 ± 3 °C, humidity 55% ± 10%, and 12 h lights on/off cycles). The Ethics Committee of the First Affiliated Hospital of Nanhua University (registration number: ChiCTR1900021723) has approved all the experiments.

Sixty mice in total were separated into six groups, (1) control, (2) sh-KDM1A, (3) circ_AFF4, (4) shKDM1A + circ_AFF4, (5) FNDC5, and (6) shKDM1A + FNDC5. BM-MSCs that infected with sh-NC and lentiviruses, sh-KDM1A, circ_AFF4 overexpression lentiviruses, sh-KDM1A plus circ_AFF4 overexpression lentiviruses, FNDC5 overexpression lentiviruses and sh-KDM1A plus FNDC5 overexpression lentiviruses cultured in OM for 1 week and seeded on bioceramic scaffolds with β-tricalcium phosphate and hydroxyapatite mixture (National Engineering Research Center for Biomaterials, Sichuan University, China) based on a previously published protocol (Liu et al. 2021). The BM-MSC-seeded scaffolds were subcutaneously placed on the dorsal side of the mice. After two months, the mice were sacrificed. Femur bone samples were harvested for the following analyses.

### Alkaline phosphatase (ALP) staining

ALP staining assay was carried out utilizing an ALP staining kit (GeFan Biotechnology, Shanghai, China). In brief, after being washed with PBS three times, cells were fixed with a fixing solution. Afterward, cells were incubated with ALP staining solution for 30 min in the dark followed by washing with PBS 3 times and visualized with a light microscope (Zeiss, Jena, Germany).

### Alizarin red S (ARS) staining

After being fixed with 4% PFA, cells were subsequently incubated with Alizarin Red S solution that was freshly prepared (Sigma-Aldrich) for about 30 min at room temperature. After 3 times wash with PBS the staining was imaged with a microscope (Zeiss).

### Actinomycin D and R treatment

BM-MSCs were seeded into six-well plates and maintained until they reach 60% confluency. Afterward, Actinomycin D (5 µg/mL) or DMSO (Sigma-Aldrich) was used to treat cells for 0, 12 and 24 h and then collected. 2 µg of total RNA was treated with RNase R (3 U/µg, Epicentre Technologies, Madison, WI, USA) for 15 min. After treatment, the RNA expression levels were analyzed by RT-qPCR.

### RNA fluorescence in situ hybridization (FISH)

After being washed 3 times with PBS, cells were treated with RNase R (Sigma-Aldrich) for 15 min followed by fixation with 4% PFA. The cells were placed onto glass slides and then dehydrated with ethanol. After hybridization, the slides were cleaned three times in 50% formamide/2 × SSC fand and incubated with circ_AFF4 and IGF2BP3 specific probes (Merck Millipore, Billerica, MA, USA) at 37 °C in standard hybridization buffer (900 mM NaCl, 20 mM Tris/HCl, 0.01% sodium dodecyl sulfate and 40% formamide). Afterward, cells treated with the DAPI (Thermo Fisher Scientific, Waltham, USA) for 15 min. The images were taken utilizing fluorescence microscopy with a 60X objective (AXIO, Zeiss).

### Immunofluorescent staining

The cells were fixed with 4% PFA for about 15 min followed by blocked with the goat serum and incubated with primary antibodies against Anti-RUNX2 (ab192256, Abcam, Cambridge, UK) at 4 °C overnight. After the secondary antibody (ab150077, Abcam) incubation, the cells were further incubated with DAPI. The images were captured under a confocal microscope (LSM710, Zeiss).

### Target prediction

The online bioinformatic tools, BLAST (https://blast.ncbi.nlm.nih.gov/Blast.cgi), was utilized to predict the binding sequence of circ_AFF4 with FNDC5, and AnimalTFDB (http://bioinfo.life.hust.edu.cn/AnimalTFDB/) was utilized to predict the interaction of KDM1A with circ_AFF4 and FNDC5.

### RNA pull-down

Biotinylated IGF2BP3 and the mutants with the mutated putative binding sites for circ_AFF4 were generated by *in vitro* transcription using TranscriptAid T7 High Yield Transcription Kit (Thermo Scientific) and purified utilizing GeneJET RNA Purification Kit (Thermo Scientific). The cell lysates that were extracted from BM-MSCs were treated with purified IGF2BP3 or mutants and with streptavidin-coated magnetic beads (Invitrogen) in order to pull down the biotin-coupled RNA complex following the user´s instructions. Magnetic beads were washed extensively. The enrichment of circ_AFF4 or IGF2BP3 was examined by RT-qPCR analysis. The bound proteins were eluted and analyzed by SDS-PAGE. The proteins were analyzed by western blot.

### RNA immunoprecipitation (RIP) assay

BM-MSCs were collected, and RIP assay was carried out utilizing an EZ-Magna RIP RNA-Binding Protein Immunoprecipitation Kit (Merck Millipore). Briefly, RIP lysis buffer was added to the cells that centrifuged at 2500 g for 10 min and then 13,000 g for 10 min. The pellets were incubated with magnetic beads conjugated to either human anti-IGF2BP3 antibody (ab225697, 1:50, Abcam), or the nonspecific anti-IgG antibody (ab172730, 1:50, Millipore) at 4 °C overnight. Afterward, protein K buffer was added for another 2 h at 4 °C. After centrifuging five times, the co-immunoprecipitated RNA was isolated and used for further RT-qPCR analysis.

### Enzyme-linked immunosorbent assay (ELISA) assay

The Irisin levels in BM-MSCs were measured using ELISA kits (NBP3-08117, Novus Biologicals, Littleton, CO, USA) based on the manufacturer’s protocol.

### Electrophoretic mobility shift assay (EMSA)

RNA EMSA assay was performed utilizing a LightShift™ Chemiluminescent RNA EMSA Kit (Thermo Fisher Scientific), based on the users´ instructions. In brief, a biotin-labeled oligonucleotide probe was purchased from Sangon Biotech (Shanghai, China) and was used to treat purified protein in a binding buffer for about 30 min. Electrophoresis on 6% nondenaturing polyacrylamide gel in 0.5× TBE buffer was used to resolve the complexes at 100 V for 1 h, which was then transferred to a nylon membrane for 30 min at 400 mA. The membrane was crosslinked, blocked and incubated with HRP-linked streptavidin for 15 min. For supershipft assay, recombinant GST-IGF2BP3 proteins were preincubated with ant-IGF2BP3 antibody (14642-1-AP, Proteintech, Chicago, IL, USA) for 20 min at 0 °C and then incubated with labeled probe for supershift assays.

### Dual-luciferase reporter assay

The assay was performed based on a reported protocol (Chen et al. 2020). Briefly, the FNDC5 wild-type sequence (FNDC5-WT) that contains the binding site of circ_AFF4 and the FNDC5 mutant sequence (FNDC5-MUT) were amplified and subsequently cloned into a psiCHECK2 vector (Promega, Shanghai, China). The mutant constructs were generated utilizing a QuickChange site-directed mutagenesis kit based on the user´s manual (Stratagene, CA, USA). Afterward, the BM-MSCs cells were co-transfected with FNDC5-WT, FNDC5-MUT and sh-circ_AFF4#1, sh_circ_AFF4#2, or lentivirus circ_AFF4 or correspondence negative controls, utilizing Lipofectamine 2000 transfection reagent (Invitrogen). The Dual-Luciferase Reporter Assay Kit (Promega) was utilized to examine the luciferase activities following 48 h incubation.

### Real-time polymerase chain reaction (RT-qPCR)

The total RNA was extracted from cells or tissues utilizing the Trizol reagent (Beyotime, Shanghai, China). 2 µg of total RNA was reversely transcribed utilizing SYBR Premix Ex Taq (Takara, Dalian, China) based on the manufacturer’s instructions. qPCR was carried out utilizing Taqman® Universal PCR mixture Kit (Thermo Fisher Scientific) based on the user’s instruction. The relative mRNA levels were quantified using the 2^−ΔΔCt^ method and GAPDH was utilized as the housekeeping gene. Primer sequences were purchased from Sangon Biotech (Shanghai, China) and their sequences were listed in Table 1.

**Table 1:** The primers in qRT-PCR

***Gene***		***Sequence***
ALP	Forward	5´-GCTGTAAGGACATCGCCTACCA-3´
	Reverse	5´-CCTGGCTTTCTCGTCACTCTCA-3´
OPN	Forward	5´-CGAGGTGATAGTGTGGTTTATGG-3´
	Reverse	5´-GCACCATTCAACTCCTCGCTTTC-3´
RUNX2	Forward	5´-CGGAATGCCTCTGCTGTTAT-3´
	Reverse	5´-TTCCCGAGGTCCATCTACTG-3´
Colla11	Forward	5´-CCTGGATGCCATCAAAGTCT-3´
	Reverse	5´-AATCCATCGGTCATGCTCTC-3´
circ_AFF4-	Forward	5´-AAAGGCCAGCATGGATCAGAA-3´
convergent	Reverse	5´-GTGATTTGGAGCGTTGATGTTC-3´
circ_AFF4-divergent	Forward	5´-GCATCGGTTTCTGGTGATGT-3´
	Reverse	5´-CGGTTCATGTTGCTTAGTTG-3´
KDM1A	Forward	5´-TCAGGAGTTGGAAGCGAATCCC-3´
	Reverse	5´-GTTGAGAGAGGTGTGGCATTAGC-3´
FNDC5	Forward	5´-GTGGTGAGCTGGGATGTTCT-3´
	Reverse	5´-GCCTGCACGTGGACTATGTA-3´
GAPDH	Forward	5´-CGACAGCAGCCGCATCTT-3´
	Reverse	5´-CCAATACGACCAAATCCGTTG-3´

### Histological analysis

Femur bone tissue from mice was fixed in 4% PFA, subsequently embedded in paraffin and cut into 5 μm sections. As previously reported (Fischer et al. 2008), the sections were stained with hematoxylin & eosin (H&E). In brief, the sections were incubated with hematoxylin (Sigma-Aldrich) for 3 min and washed for 1 min. Afterward, these sections were stained with eosin (Sigma-Aldrich) for 45s. The sections were visualized utilizing a microscope after dehydrating and mounting.

### Immunohistochemistry (IHC) staining

Bone tissues were fixed in 4% PFA and were subsequently embedded in paraffin. IHC staining was performed on 5 μm slides from embedded blocks. Slides were stained using the protocol as described previously (Koukourakis et al. 2005). The sections were incubated with a RUNX2 primary antibody (ab236639, Abcam), for 1 h at RT and followed by incubation with a secondary antibody (ab150077, Abcam). The sections were counterstained with hematoxylin and visualized under a Zeiss confocal microscope.

### Masson staining

Trichrome Stain Kit (Sigma-Aldrich) was used for Masson staining based on the manufacturer’s protocol. The images were taken using a light microscope.

### Western blot

Total cellular protein was isolated from the cells using the radio-immunoprecipitation assay (RIPA) buffer (Beyotime) containing protease inhibitors at 4 ºC for 30 min. Protein concentrations were measured with BCA protein, separated by SDS-PAGE (30 µg/well), and transferred to polyvinylidene fluoride membranes and blocked. The membranes were incubated with primary antibodies including ALP (ab229126, Abcam), OPN (ab283656, Abcam), RUNX2 (ab23981, Abcam), Colla1 (ab260043, Abcam), and KDM1A (ab62582, Abcam). Afterward, these membranes were washed and followed by incubation with a secondary antibody (ab7090, Abcam). Eventually, an ECL kit (Millipore) was utilized to visualize the protein bands.

### Chromatin immunoprecipitation (ChIP)

The EZ-ChIP kit (Upstate, Lake Placid, NY, USA) was used to perform the ChIP assay based on a previously published protocol (Milne et al. 2009). In brief, BM-MSCs were resuspended in SDS lysis buffer and then sheared by sonication. The chromatin fragments were immunoprecipitated with antibodies against KDM1A (ab129195, Abcam), H3K9me3 (ab8898, Abcam) and the purified DNA was analyzed using RT-qPCR.

### Statistical analysis

All statistical analysis was performed using GraphPad Prism 5.0. Two groups were compared using an unpaired student t-test. One-way analysis of variance (ANOVA) followed by the Turkey post hoc test was applied in cases of comparison among multiple groups. *P* < 0.05 was considered significant. All data are shown as mean ± standard deviation. At least three independent experiments were carried out.

## Results

### Circ_AFF4 and KDM1A were significantly increased in BM-MSCs upon osteoinduction

**Fig. 1:**
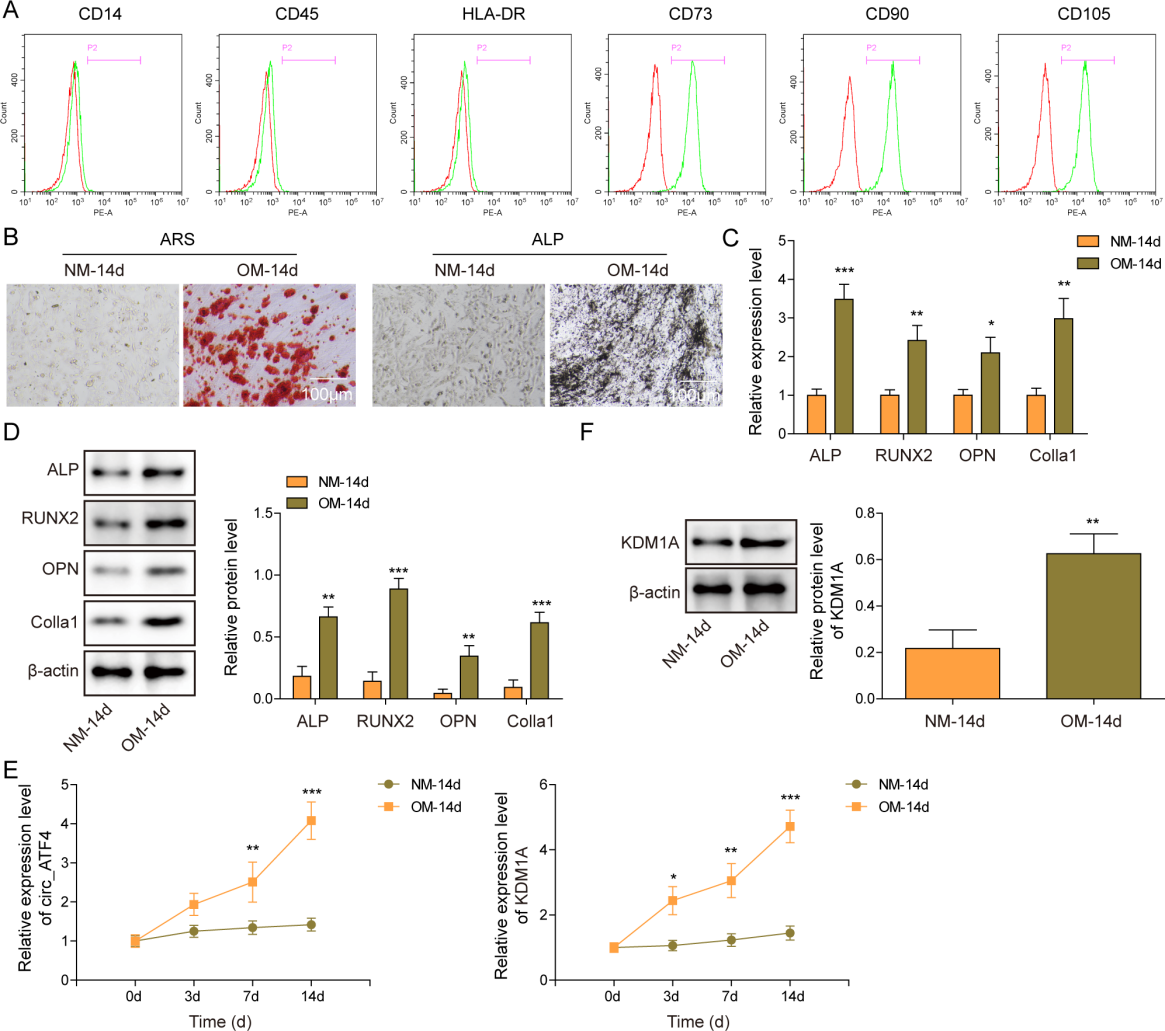
**Circ_AFF4 and KDM1A were significantly increased in BM-MSCs upon osteoinduction** (**A**) Protein expression of cell surface markers, CD14, CD45, HLA-DR, CD73, CD90 and CD105, in BM-MSCs was assessed by FACS. (**B**) Representative images of ARS and ALP staining of BM-MSCs cultured in osteogenic medium (OM) or normal medium (NM) for 14-days. Scar bar = 100 μm. Relative gene (**C**) and protein (**D**) expression of osteogenic markers, ALP, RUNX2, OPN and Colla 1, in BM-MSCs cultured in OM or NM for 14-days, were detected by RT-qPCR and western blot, respectively. (**E**) Relative gene expression of circ_AFF4 and KDM1A were assessed by RT-qPCR in BM-MSCs cultured in OM or NM after 0, 3, 7 and 14 days. (**F**) Relative protein expression of KDM1A was detected by western blot in BM-MSCs cultured in OM or NM after 14 days. *p < 0.05, **p < 0.001 and ***p < 0.001. Each experiment was performed at least three times independently

As shown by FACS, the protein expression levels of surface markers CD90, CD73 and CD105 were detected in the BM-MSCs while CD45, HLA-DR and CD14 protein expression levels were not examined in BM-MSCs (Fig. 1 A). The BM-MSCs were next cultured in an osteogenic medium (OM) for 14 days to induce osteogenic differentiation. As observed in ARS and ALP staining images, the BM-MSCs cultured in OM displaced positive staining (Fig. 1B), suggesting the efficient osteogenic differentiation induction. Consistently, the gene and expression levels of osteogenesis markers, ALP, RUNX2, OPN and Colla1, were significantly upregulated in BM-MSCs cultured in OM when compared to those cultured in normal medium (NM) (Fig. 1 C and 1D). The relative gene expression of circ_AFF4 and KDM1A were significantly increased in BM-MSCs that were treated with OM after 3-day, 7-day and 14-day in a time-dependent manner (Fig. 1E). The protein expression of KDM1A was also enhanced in OM-treated BM-MSCs after 14 days (Fig. 1 F).

Altogether, circ_AFF4 and KDM1A expression was significantly enhanced in BM-MSCs upon osteoinduction.

### Characterization of circ_AFF4 in BM-MSCs

We next characterize circ_AFF4 by Sanger sequencing (Supplementary Fig. 1 A), RT-qPCR (Supplementary Fig. 1B), RNase R (Supplementary Fig. 1 C) and Actinomycin D (Supplementary Fig. 1D) treatment in BM-MSCs. Circ_AFF4 was derived from exons 1–6 of AFF4 located at chr5: 132,262,812–132,272,885, with a spliced sequence length of 1054 nt (Supplementary Fig. 1 A). The complementary DNA products of circ_AFF4 were amplified using PCR and examined by Sanger sequence. We next designed divergent primers to amplify linear AFF4 mRNA and circ_AFF4 to verify head-to-tail splicing. As indicated in Supplementary Fig. 1B, the circ_AFF4 can only be amplified in cDNA. Circ_AFF4 showed resistance to RNase digestion indicating that it harbors a circular structure (Supplementary Fig. 1 C). In contrast, linear AFF4 was digested with RNase. There is an enrichment of circ_AFF4 compared to the associated linear transcript after Actinomycin D treatment in a time-dependent manner (Supplementary Fig. 1D). Nuclear and cytoplasmic fractionation and FISH experiments indicated that the majority of circ_AFF4 was shown in the cell cytoplasm but some were also displayed in the nucleus (Supplementary Fig. 1E-1 F).

Taken together, circ_AFF4 is a stable and abundant circRNA in BM-MSCs.

### Knockdown of circ_AFF4 suppresses BM-MSC osteogenic differentiation

**Fig. 2:**
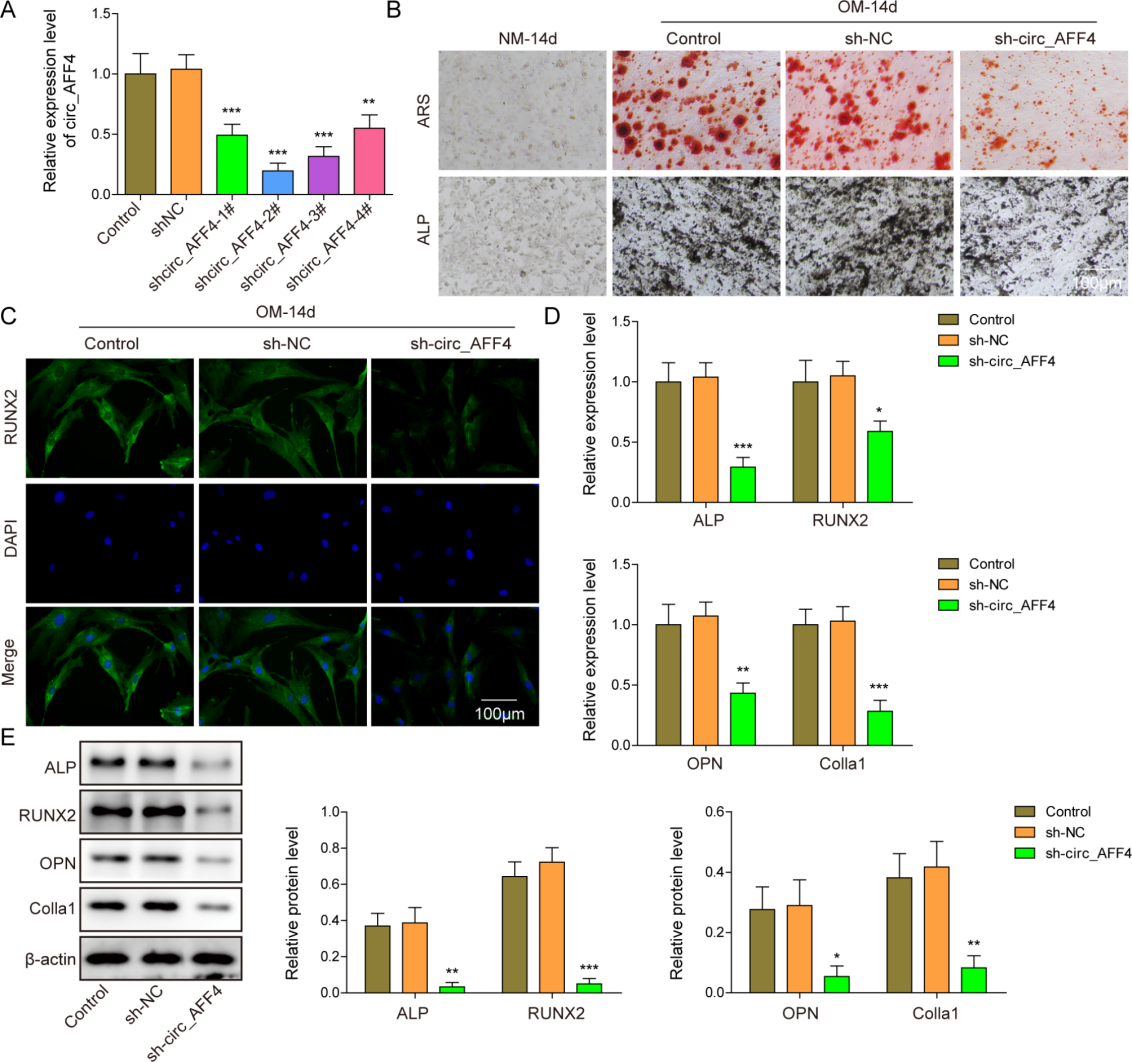
**Knockdown of circ_AFF4 suppresses BM-MSC osteogenic differentiation** (**A**) BM-MSCs were transfected with shcirc_AFF4_1–4# or shNC and the expression level of circ_AFF4 in shcirc_AFF4 or shNC transfected BM-MSCs was detected using RT-qPCR. BM-MSCs that were transfected with shcirc_AFF4 or shNC were cultured in OM or NM for 14 days. Afterward, different assays were performed to assess the osteogenic differentiation. (**B**) Representative images of ARS and ALP staining. Scar bar = 100 μm. (**C**) Representative RUNX2 immunofluorescence staining image. Scar bar = 100 μm. Gene and protein expression of osteogenic markers, ALP, OPN, RUNX2 and Colla1 were detected by (**D**) RT-qPCR and (**E**) western blot. * *p* < 0.05, ** *p* < 0.001 and *** *p* < 0.001. Each experiment was performed at least three times independently

To explore the role of circ_AFF4 in BM-MSC osteogenic differentiation, we knockdown circ_AFF4 in BM-MSCs using shcirc_AFF4. As shown in Fig. 2 A, the expression level of circ_AFF4 is significantly reduced in BM-MSCs after being transfected with shcirc_AFF4_1#, shcirc_AFF4_2#, shcirc_AFF4_3# and shcirc_AFF4#. Considering that shcirc_AFF4_2# with highest transfection efficiency, it was selected as the follow-up study. After successfully knockdown of circ_AFF4, BM-MSCs were cultured in OM for another 14 days to examine the effect of circ_AFF4-depletion on osteogenic differentiation. circ_AFF4-depleted BM-MSCs displayed diminished osteogenic differentiation and calcium deposits after 14-day culture in OM compared to the non-treated group and shNC group as observed in ALP and ARS staining images (Fig. 2B). RUNX2 protein synthesis in OM-treated BM-MSCs was inhibited by silencing of circ_AFF4 (Fig. 2 C). Furthermore, as assessed by RT-qPCR (Fig. 2D) and western blot (Fig. 2E), the osteogenic differentiation factors, including ALP, Runx2, OPN and Colla1, were reduced in circ_AFF4-depleted BM-MSCs after 14-day culture in OM in relation to the non-treated group and the shNC group.

Collectively, Knockdown of circ_AFF4 inhibits osteogenic differentiation of BM-MSCs.

### Circ_AFF4 interacts with IGF2BP3 and stabilizes FNDC5 mRNA

**Fig. 3:**
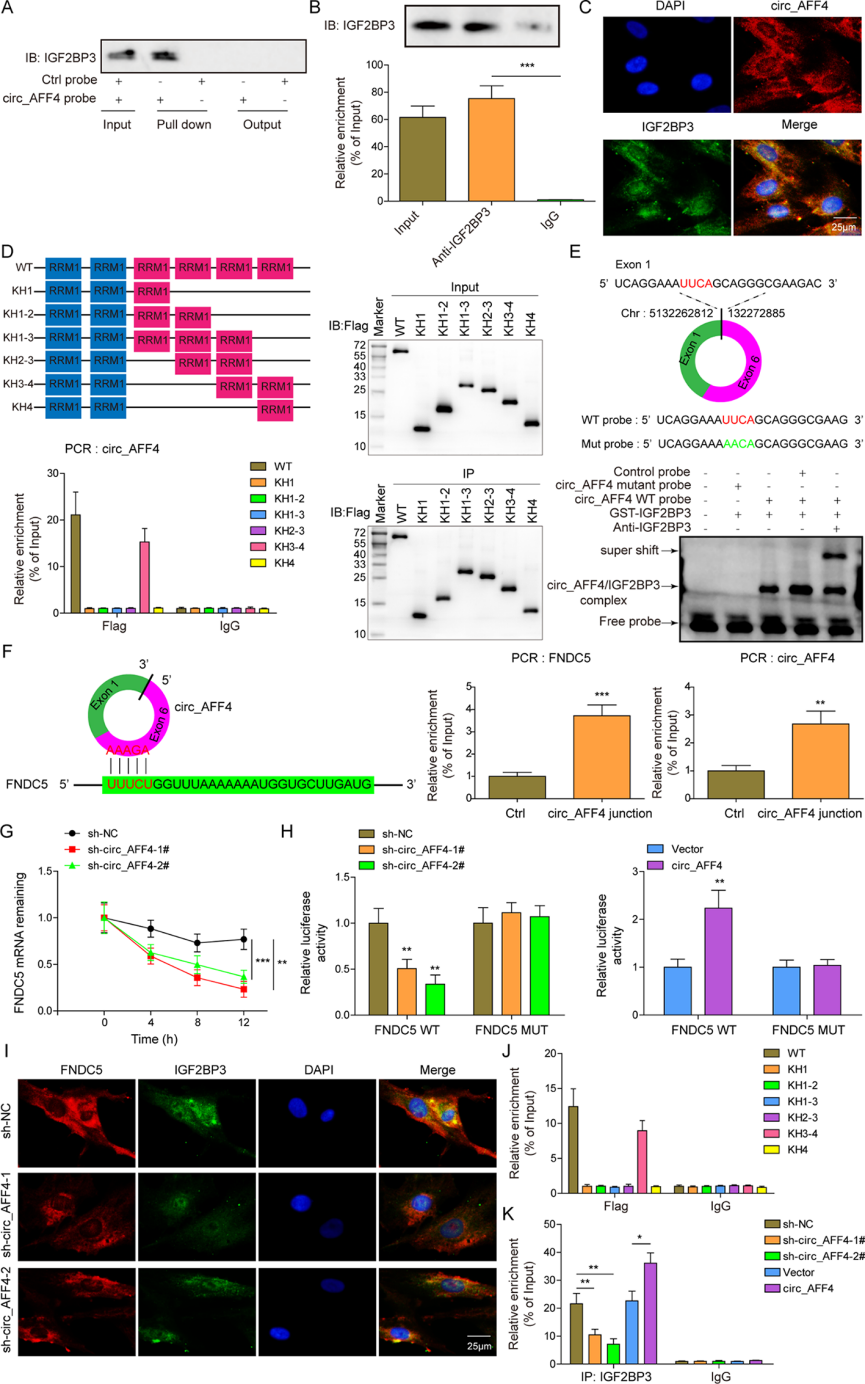
**Circ_AFF4 interacts with IGF2BP3 and stabilizes FNDC5 mRNA** (**A**) Immunoblot analysis of IGF2BP3 after pulldown assay showing it interacts with circ_AFF4-IGF2BP3. (**B**) RIP assay indicating the interaction of IGF2BP3 with circ_AFF4 in BM-MSCs. (**C**) FISH assay showing the localization of circ_AFF4 and IGF2BP3 protein in the cytoplasm in BM-MSCs. Scar bar = 25 μm. (**D**) Upper left, RNA-binding domains within IGF2BP3 protein and a summary of IGF2BP3 truncations were shown in schematic structures. Bottom Left, relative enrichment of circ_AFF4 indicating the association of circ_AFF4 with truncated IGF2BP3 compared to the control (IgG). Right, immunoblot analysis with anti-FLAG of BM-MSCs transfected with plasmids encoding FLAG-tagged WT or truncated IGF2BP3. (**E**) Top, UUCA motif located at exon 1-exon 6 junction site of circ_AFF4 and the RNA probe for RNA-EMSA assay were shown with a schematic illustration. Bottom, RNA-EMSA assay showed the binding ability of purified IGF2BP3 with biotin-labeled oligonucleotides containing UUCA motif from circ_AFF4. (**F**) Left, sequence BLAST analysis indicating that circ_AFF4 directly targets the 3′UTR of FNDC5. Right, relative enrichment of FNDC5 and circ_AFF4 showing the association of FNDC5 and circ_AFF4 junction compared to control. (**G**) Circ_AFF4 knockdown significantly reduced FNDC5 enrichment in BM-MSCs. (**H**) Luciferase activity of luciferase reporter gene with FNDC5-WT or FNDC5-mut in circ_AFF4-overexpressed or circ_AFF4-knockdown BM-MSCs. (**I**) The co-localization of IGF2BP3 and FNDC5 was shown FISH image. Scar bar = 25 μm. (**J**) Relative enrichment of FNDC5 indicating the association of FNDC5 with truncated IGF2BP3 protein. (**K**) RIP assay showing the association of IGF2BP3 with FNDC5 upon circ_AFF4 knockdown or overexpression. * *p* < 0.05, ** *p* < 0.001 and *** *p* < 0.001. Each experiment was performed at least three times independently.

As it was predicted by the computational prediction tool predicted that circ_AFF4 may potentially bind to IGF2BP3, we used RNA pull-down assay, RIP assay and FISH assay to confirm this interaction. It was observed by RNA pull-down assay that circ_AFF4 was pull-downed with IGF2BP3 protein (Fig. 3 A). RIP assay further proved the interaction between circ_AFF4 and IGF2BP3 (Fig. 3B). The FISH assay showed the co-localization of circ_AFF4 and IGF2BP3 expressed in the cell cytoplasm (Fig. 3 C). To identify which domain of IGF2BP3 interacts with circ_AFF4, IGF2BP3 mutants with truncation of each KH domain was constructed. It was shown that the KH3-4 di-domain of IGF2BP3 was bound to circ_AFF4 (Fig. 3D). To find the motif within circ_AFF4 required for IGF2BP3 recruitment, it is predicted that IGF2BP3 bound to exon 1-exon 6 junction site of circ_AFF4 and UUCA motif was identified as a putative binding motif of IGF2BP3 (Fig. 3E, top). Next, *in vitro* RNA-EMSA was performed to further confirm this. As indicated by supershift experiments, once mutated the UUCA binding motif of circ_AFF4, the interaction ability of IGF2BP3 with circ_AFF4 was reduced (Fig. 3E, bottom). These data suggest that IGF2BP3 directly binds to the UUCA motif of circ_AFF4 via the KH3-4 di-domain.

It was shown by previous studies that IGF2BP3 or IGF2BP3/circRNA complex stabilizes mRNA (Wang et al. 2022). Therefore, we next investigated if IGF2BP3/circ_AFF4 stabilizes downstream targets. By utilizing BLAST analysis (https://blast.ncbi.nlm.nih.gov/Blast.cgi), we identified that the AAAGA site in circ_AFF4 may directly bind to the 3´UTR of FNDC5 with AU-Rich Elements (Fig. 3 F). RNA pull-down assay indicated the interaction between circ_AFF4 and FNDC5. Knockdown of circ_AFF4 significantly decreased FNDC5 mRNA stability in a time-dependent manner (Fig. 3G). To elucidate the role of circ_AFF4/FNDC5 complex formation on FNDC5 mRNA stabilization, luciferase reporter genes that contain FNDC5-WT or FNDC5-mut were constructed. Circ_AFF4 depletion significantly suppressed the luciferase activity of FNDC5-WT but not of FNDC5-mut in BM-MSCs (Fig. 3 H). In contrast, overexpression of circ_AFF4 enhanced the luciferase activity of FNDC5-WT but not of FNDC5-mut. RNA-FISH assay indicated that IGF2BP3 and FNDC5 were co-localized in the cell cytoplasm (Fig. 3I). The absence of circ_AFF4 reduced the co-localization of the IGF2BP3 and FNDC5 complex. RIP assay indicated that the interaction of IGF2BP3 with circ_AFF4 required KH3-4 di-domain of IGF2BP3 (Fig. 3 J). Silence of circ_AFF4 significantly decreased the IGF2BP3 and FNDC5 interaction, whereas circ_AFF4 overexpression showed an opposite effect (Fig. 3 K). Taken together, these data revealed that IGF2BP3 binds to the UUCA motif of circ_AFF4 via the KH3-4 di-domain. Furthermore, circ_AFF4 plays a crucial role in IGF2BP3 and FNDC5 interaction and increases the stability of FNDC5 by formatting a circ_AFF4, IGF2BP3 and FNDC5 RNA-protein complex.

### Induction of circ_AFF4 on osteogenic differentiation via FNDC5/Irisin was counteracted by IGF2BP3 downregulation

**Fig. 4:**
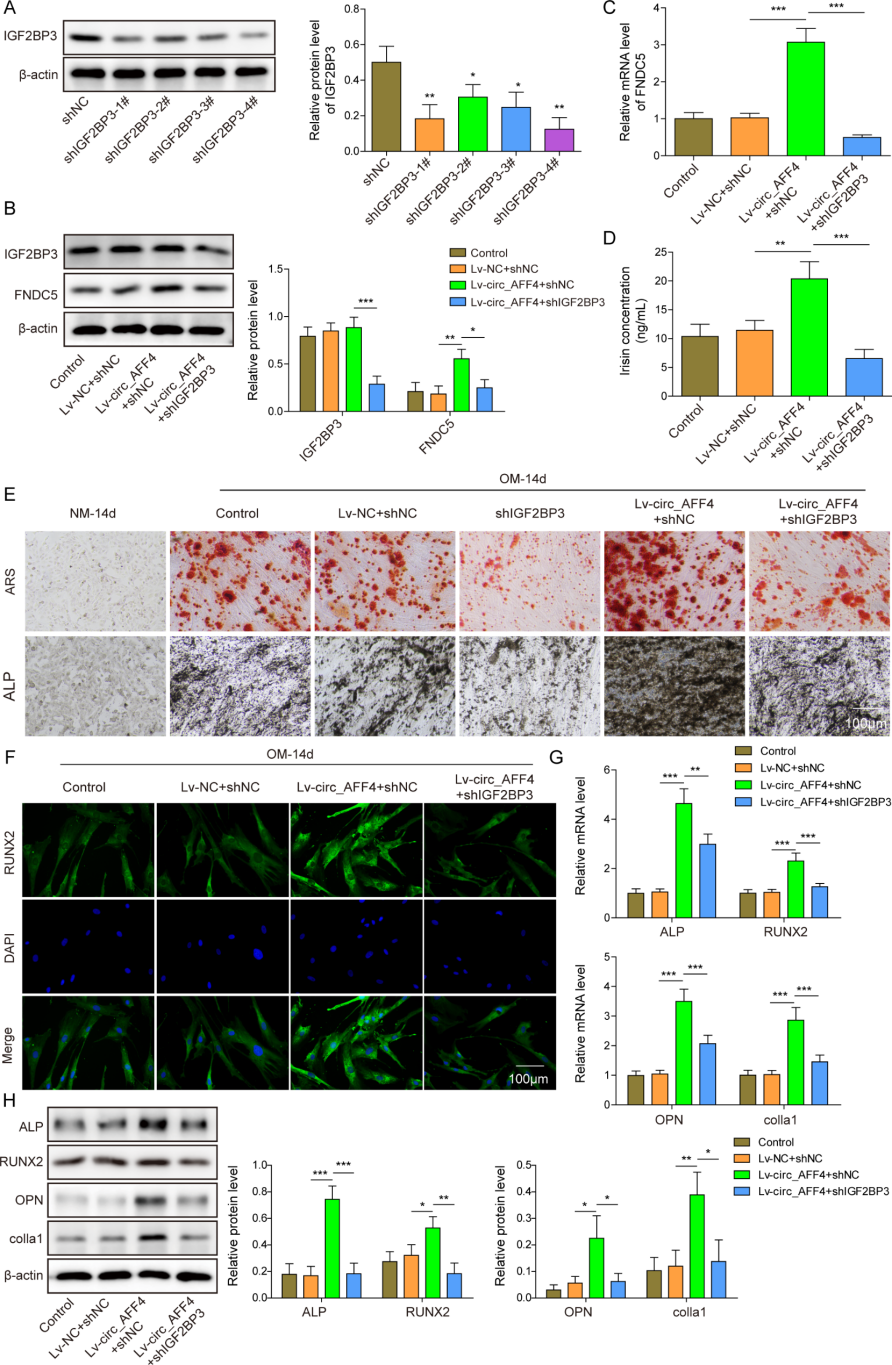
**Promotion of circ_AFF4 on osteogenic differentiation via FNDC5/Irisin was counteracted by IGF2BP3 downregulation** (**A**) BM-MSCs were transfected with shIGF2BP3_1–4# or shNC and the protein expression level of IGF2BP3 in shIGF2BP3_1–4# or shNC transfected BM-MSCs was detected using western blot. BM-MSCs that transfected with lentivirus-circ_AFF4 (lv-circ_AFF4) and shNC or shIGF2BP3 and lv-circ_AFF4 or shNC and lentivirus-NC (lv-NC) were cultured in OM or NM for 14 days. Gene and protein expression of IGF2BP3 and FDNC5/Irisin were assessed by western blot (**B**), RT-qPCR (**C**) and ELISA(**D**). Afterward, different assays were performed to assess the osteogenic differentiation. (**E**) Representative images of ARS and ALP staining. Scar bar = 100 μm. (**F**) Representative RUNX2 immunofluorescence staining image. Scar bar = 100 μm. Gene and protein expression of osteogenic markers, ALP, OPN, RUNX2 and Colla1, were detected by (**G**) RT-qPCR and (**H**) western blot. *p < 0.05, **p < 0.001 and ***p < 0.001. Each experiment was performed at least three times independently.

To thoroughly understand the role of circ_AFF4 and IGF2BP3 in BM-MSC osteogenic, we firstly transfected BM-MSCs with lentiviruses harboring circ_AFF4 or shIGF2BP3 for a stable overexpression or knockdown followed by and osteogenesis capacity evaluation after culturing in OM for 14 days. Transfection of BM-MSC with shIGF2BP3-1#, shIGF2BP3-2#, shIGF2BP3-3#, shIGF2BP3-4# resulted in a decrease in IGF2BP3 expression (Fig. 4 A). Transfection with shIGF2BP3-4# reduced the IGF2BP3 expression most, which was used for the following experiments. Circ_AFF4 overexpression did not alter IGF2BP3 expression but significantly induced FNDC5 expression and Irisin concentration in BM-MSCs that were treated with OM after 14 days (Fig. 4B and D). Nevertheless, the knockdown of IGF2BP3 reversed the promotion effect of circ_AFF4 overexpression on FNDC5 expression and Irisin concentration. IGF2BP3-depleted BM-MSCs cultured in OM for 14 days displayed decreased staining intensity of both ARS and ALP compared to the control group (Fig. 4E). The staining intensity of ARS and ALP, which was decreased in circ_AFF4 overexpressed cells, clearly declined upon sh-IGF2BP3 transfection (Fig. 4E), Circ_AFF4 overexpression induced RUNX2 positive BM-MSC number (Fig. 4 F). However, the increase of RUNX2 positive cells was reversed by the knockdown of IGF2BP3. Consistently, overexpression of circ_AFF4 led to a significant increase in both gene and protein expression of osteogenesis-related factors, including ALP, Runx2, OPN and Colla1, which were counteracted by IGF2BP3 depletion (Fig. 4G H).

Collectively, Circ_AFF4 and IGF2BP3 regulate BM-MSC osteogenic differentiation via FNDC5/Irisin.

### Knockdown of KDM1A suppresses osteogenic differentiation of BM-MSCs

**Fig. 5:**
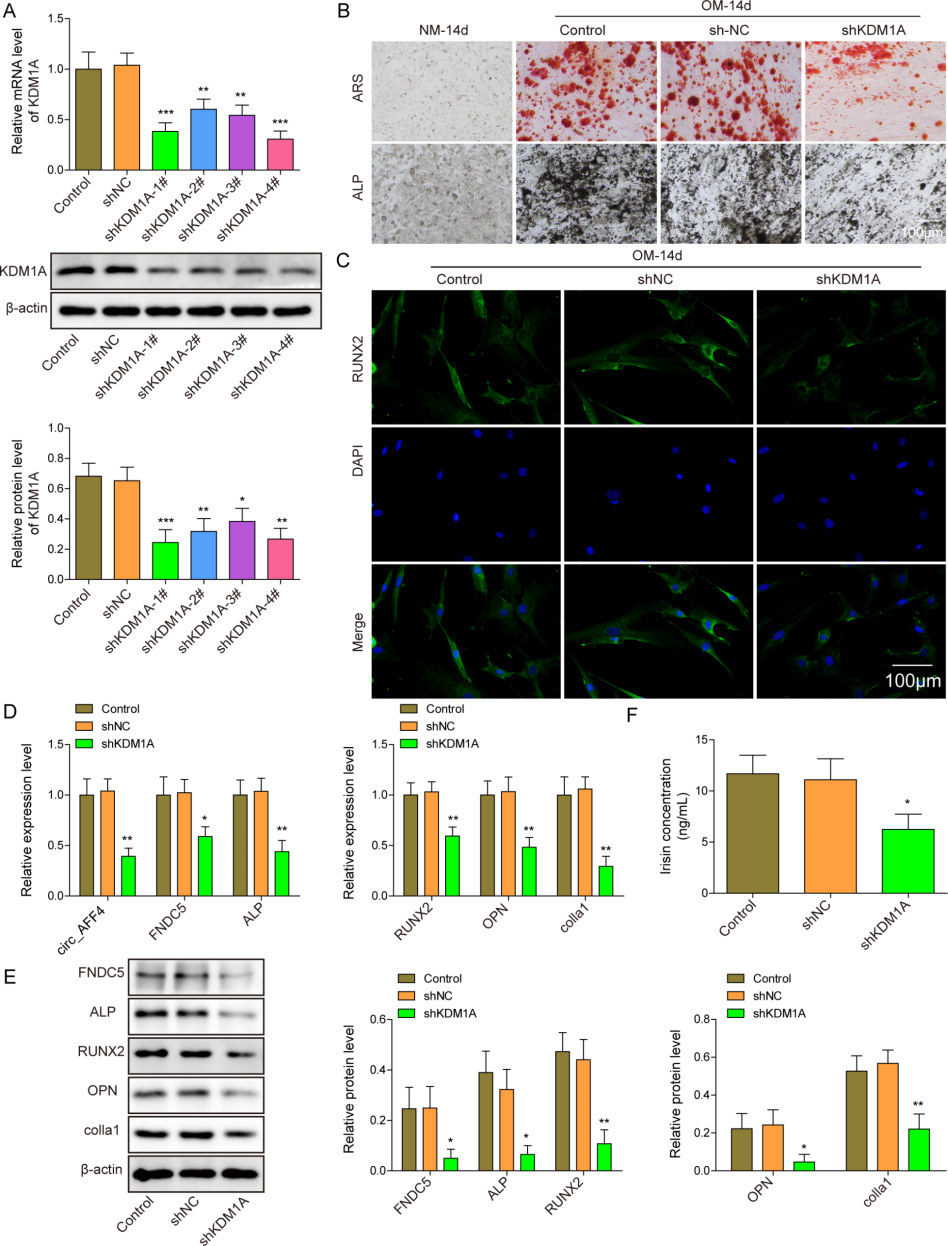
**Knockdown of KDM1A suppresses osteogenic differentiation of BM-MSCs** (**A**) BM-MSCs were transfected with shKDM1A_1–4# or shNC and the expression level of KDM1A in shKDM1A_1–4# or shNC transfected BM-MSCs was detected using western blot. BM-MSCs that were transfected with shKDM1A or shNC were cultured in OM or NM for 14 days. Afterward, different assays were performed to assess the osteogenic differentiation. (**B**) Representative images of ARS and ALP staining. Scar bar = 100 μm. (**C**) Representative RUNX2 immunofluorescence staining image. Scar bar = 100 μm. Gene and protein expression of osteogenic markerslivergent, ALP, OPN, RUNX2 and Colla1, and circ_AFF4, FNDC5 were detected by (**D**) RT-qPCR and (**E**) western blot. (**F**) ELISA detected Irisin expression. *p < 0.05, **p < 0.001 and ***p < 0.001. Each experiment was performed at least three times independently.

A recent study showed that KDM1A mediated osteogenic differentiation of MSCs (Wang et al. 2018). BM-MSCs were transfected with shKDM1A to knockdown KDM1A. As shown in Fig. 5 A, the KDM1A gene and protein expression were significantly reduced in all shKDM1A transfected BM-MSCs. shKDM1A-1# showed the highest efficiency and was thereby chosen for further experiments. The downregulation of KDM1A attenuated ALP and ARS staining and decreased the number of RUNX2 positive cells as indicated by ALP and ARS staining images and immunofluorescent images, respectively (Fig. 5B C). KDM1A knockdown further reduced circ_AFF4, FNDC5, osteogenesis-related factors, including ALP, Runx2, OPN and Colla1, and Irisin concentration in BM-MSCs after being cultured in OM for 14 days (Fig. 5D F).

Taken together, knockdown of KDM1A inhibits the osteogenic differentiation capacity of BM-MSCs.

### KDM1A directly binds to circ_AFF4 and FNDC5 to promote FNDC5/Irisin expression

**Fig. 6:**
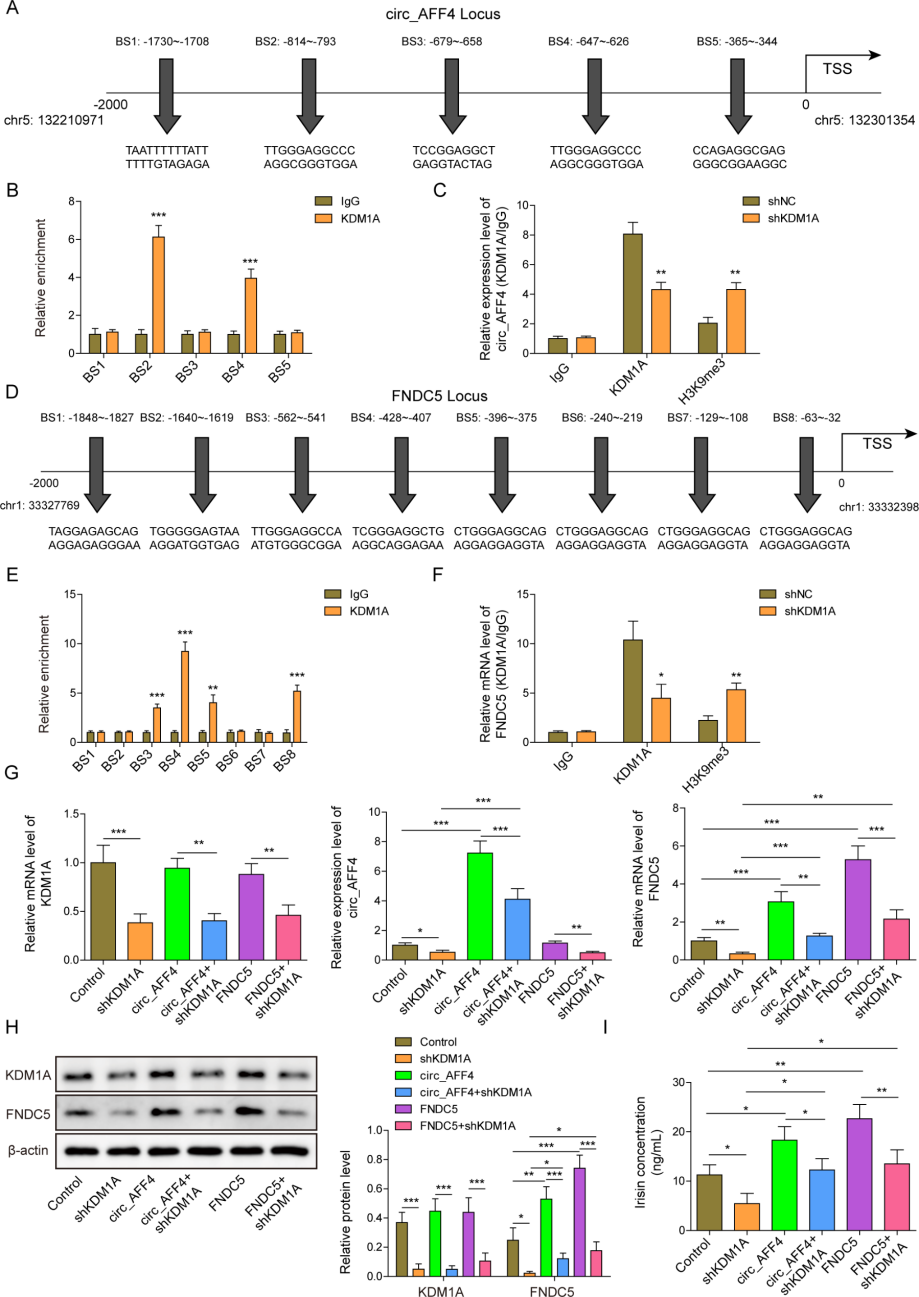
**KDM1A directly binds to circ_AFF4 and FNDC5 to promote FNDC5/Irisin expression** (**A**) Schematic illustration showing binding sites between circ_AFF4 promoter and KDM1A predicted by AnimalTFDB, (**B**) ChIP assay showing the enrichment of BS1-5 sites of circ_AFF4 promoter in complexes precipitated with antibody against KDM1A. (**C**) ChIP assay showing the impact of KDM1A knockdown on KDM1A/circ_AFF4 promoter interaction. (**D**) Schematic illustration showing binding sites between FNDC5 promoter and KDM1A predicted by AnimalTFDB, (**E**) ChIP assay showing the enrichment of BS1-8 sites of FNDC5 promoter in complexes precipitated with antibody against KDM1A. (**F**) ChIP assay showing the impact of KDM1A knockdown on KDM1A/FNDC5 promoter interaction. Circ_AFF4, FNDC5 and KDM1A were detected by (**G**) RT-qPCR and (**H**) western blot. (**I**) ELISA detected Irisin expression. *p < 0.05, **p < 0.001 and ***p < 0.001. Each experiment was performed at least three times independently

The computational prediction tool, AnimalTFDB, predicted that there might be five binding sites between KDM1A and circ_AFF4 promoter (Fig. 6 A). ChIP assay demonstrated the enrichment of BS2 site and BS4 site in complexes precipitated with antibody against KDM1A (Fig. 6B). KDM1A could demethylate H3K9me3 and upregulated the expression of genes in MSCs, in our study, and we found knockdown of KDM1A reduced the KDM1A/circ_AFF4 promoter interaction, whereas H3K9me3 significantly increased enrichment of circ_AFF4 promoter in KDM1A immunoprecipitated fractions (Fig. 6 C). Similarly, AnimalTFDB, predicted that there might be eight binding sites between KDM1A and FNDC5 promoter (Fig. 6D). As shown by ChIP assay result, the enrichment of BS3, BS4, BS5 and BS8 sites were observed in complexes precipitated with antibodies against KDM1A (Fig. 6E). Downregulation of KDM1A decreased the KDM1A/FNDC5 promoter interaction, whereas H3K9me3 significantly enhanced enrichment of FNDC5 promoter in KDM1A immunoprecipitated fractions (Fig. 6 F). Altogether, these data demonstrated that KDM1A promotes circ_AFF4 and FNDC5 expression via reducing H3K9me3.

To further confirm the role of KDM1A in circ_AFF4 or FNDC5 mediated osteogenic differentiation, BM-MSCs were transfected with shKDM1A, lentiviruses harboring circ_AFF4, lentiviruses harboring circ_AFF4 plus shKDM1A, lentiviruses harboring FNDC5 or lentiviruses harboring FNDC5 plus shKDM1A to knockdown KDM1A and overexpress circ_AFF4 or FNDC5. The transfection was performed successfully as shown in Fig. 6G H. Circ_AFF4 or FNDC5 overexpression significantly increased Irisin concentration, which was reversed by KDM1A depletion (Fig. 6I).

These data suggest that KDM1A locates at upstream of circ_AFF4 and FNDC5 and binds to circ_AFF4 and FNDC5 to promote Irisin expression.

#### KDM1A enhances bone formation by regulating circ_AFF4 and FNDC5 *in vivo*

To identify the *in vivo* role of KDM1A, circ_AFF4 and FNDC5 in bone formation, we seeded BM-MSCs transfected with lentiviruses harboring circ_AFF4, lentiviruses harboring FNDC5 or shKDM1A or their correspondence controls on scaffold material circ_AFF4 and then implanted in mice. As observed in ALP and ARS staining images, bone samples from mice with circ_AFF4 overexpression or FNDC5 overexpression displayed increased bone cell differentiation and calcium deposits (Supplementary Fig. 2 A). Knockdown of KDM1A reversed circ_AFF4 or FNDC5 overexpression-induced osteogenic differentiation and calcium deposits. Similarly, new bone regeneration was increased in circ_AFF4 or FNDC5 overexpressed BM-MSC treated mice as revealed by H&E staining (Supplementary Fig. 2B), which was reversed by KDM1A knockdown. Masson’s trichrome staining indicated that overexpression of circ_AFF4 or FNDC5 induced collagen deposition at the new bone formation area and promoted new bone growth (Supplementary Fig. 2 C). However, the promotion effect of circ_AFF4 or FNDC5 overexpression was diminished by KDM1A depletion. RUNX2 protein synthesis was promoted by overexpression of circ_AFF4 or FNDC5, which was reversed by silencing of KDM1A (Supplementary Fig. 2D). Additionally, downregulation of KDM1A attenuated circ_AFF4 or FNDC5 overexpression-induced osteogenic factors both in gene and protein levels (Supplementary Fig. 2E and 2 F). Overexpression of circ_AFF4 or FNDC5 has no impact on KDM1A protein expression.

Altogether, circ_AFF4 or FNDC5 induces new bone formation *in vivo*. Nevertheless, this promotion effect is reversed by KDM1A depletion, indicating that KDM1A regulates bone formation via circ_AFF4 and FNDC5.

## Discussion

In this study, we demonstrated the importance of circ_AFF4 in osteogenesis both *in vitro* and *in vivo*. We showed that circ_AFF4 was increased during osteoinduction of BM-MSCs. Increased circ_AFF4 expression promotes the stability of FNDC5 mRNA by generating a circ_AFF4/IGF2BP3/FNDC5 ternary complex to enhance osteogenic differentiation of BM-MSCs. This study further revealed that KDM1A locates at upstream of circ_AFF4 and induces the expression of circ_AFF4, which enhances the downstream protein IGF2BP3/FNDC5 and further increases KDM1A expression. As far as we know, this is the first time demonstrating that the KDM1A/circ_AFF4/FNDC5 positive axis promotes osteogenesis via FNDC5/Irisin. This *in vitro* data was further confirmed with *in vivo* model.

Circ_AFF4 was demonstrated to be involved in mediating osteogenesis (Lin et al. 2021; Mi et al. 2019; Liu et al. 2021). Another study documented that circ_AFF4 enhanced osteoblast proliferation by acting as a sponge of miR-7223-5p *in vitro* and promoted fracture healing *in vivo* (Mi et al. 2019). Consistently, we demonstrated that circ_AFF4 was upregulated in osteoinduction of BM-MSCs in this study. Knockdown of circ_AFF4 inhibited the osteogenesis of BM-MSCs, suggesting that circ_AFF4 plays a critical role in the osteogenic differentiation of BM-MSCs. The pull-down experiments, RIP assay and FISH indicated that IGF2BP3 was a downstream target of circ_AFF4, which directly bound FNDC5. Circ_AFF4 overexpression promoted expression of FNDC5/Irisin, while knockdown of IGF2BP3 reversed circ_AFF4 overexpression induced FNDC5/Irisin and thereby inhibited BM-MSC osteogenesis. Previous studies indicated that IGF2BP3 may act as a diagnostic biomarker for various cancers due to its abnormal expression in tumors (Mancarella and Scotlandi 2019). However, the functional role of IGF2BP3 in osteogenesis was barely reported. To the best of our knowledge, this was the first time reported that IGF2BP3 mediated BM-MSC osteogenic differentiation via FNDC5/Irisin. Other studies also documented that FNDC5/Irisin was involved in bone formation (Deng et al. 2020). For instance, silence of FNDC5/Irisin in mice resulted in delayed bone development and a lower bone density compared to normal mice (Deng et al. 2020). Osteogenic differentiation of MSCs was also inhibited in FNDC5/Irisin deficiency mice. Another study from Liu et al. (2021). Circ_AFF4 can mediate osteogenesis via miR-135a-5p/ FNDC5/Irisin axis per previous study (Liu et al. 2021) or IGF2BP3/ FNDC5/Irisin according to this study. Interestingly, miRNAs were reported to be able to bind IGF2BP3 (Hu et al. 2020). miR-125a-5p targeted IGF2BP3 in advanced gastric cancer to inhibit the cancer´s progression (Hu et al. 2020). Therefore, we speculated that there could be two regulatory mechanisms, either miR-135a-5p potentially also interacts IGF2BP3 to regulate FNDC5/Irisin or miR-135a-5p does not bind IGF2BP3 and these two axes function independently. Future studies may focus on further investigating the relationship of miR-135a-5p and IGF2BP3 in regulating BM-MSC osteoinduction. - It is acknowledged that circRNAs exert their biological functions via three mechanisms (Zhou et al. 2020). Circ_RNAs act as sponges of miRNAs via the binding sites to regulate miRNA activities or are translated as encoded proteins or regulate gene expression at the splicing and transcription levels (Panda 2018; Lei et al. 2020; Shao et al. 2021; Chen et al. 2019). Specifically, circ_NSUN2 was shown to enhance mRNA HMGA2 stability by forming an RNA-protein ternary complex (Chen et al. 2019). In this present study, we demonstrated that circ_AFF4, IGF2BP3 and FNDC5 interacted with each other to form a circ_AFF4/IGF2BP3/FNDC5 ternary complex and thereby enhanced FNDC5 mRNA stability, which further confirmed this novel discovery that circRNAs can increase mRNA stability via interacting with mRNA-binding proteins. This may advance our knowledge of how circRNAs employ their biological functions.

This study also revealed the crucial role of KDM1A in circ_AFF4 mediating BM-MSC osteogenesis. KDM1A was elevated in BM-MSCs that were treated with OM for 14 days and KDM1A promoted bone formation *in vivo*. Nevertheless, a publication showed that KDM1A knockdown suppressed ALP activity and mineralization *in vitro* but promoted osteogenesis-related factors *in vitro* and *in vivo* (Wang et al. 2018). Another study by Sun et al. indicated that KDM1A acted as a key regulator of osteoblast differentiation and KDM1A deficiency led to increased bone formation (Ge et al. 2014). These contradictory results may indicate the dynamic role of KDM1A during osteoinduction. Our study further suggested the positive regulatory axis of KDM1A/circ_AFF4/FNDC5 in regulating osteogenesis. ChIP assay suggested that KDM1A was directly bound to circ_AFF4 and FNDC5. Suppression of KDM1A significantly inhibited circ_AFF4 and FNDC5 expression via H3K9me3 demethylation and thereby reversed circ_AFF4/FNDC5 overexpression-induced bone formation.

This study has two limitations. First, the animal experiments in this study did not include any controls for exercise level in mice but physical activity is an important factor of Irisin expression. Secondly, the sample sizes for this study, especially *in vitro* experiments, were relatively limited since most of the *in vitro* study data were collected from only three independent studies.

In conclusion, for the first time, we reported the KDM1A/circ_AFF4/FNDC5 axis during BM-MSC osteoinduction. We further uncovered the critical role of circ_AFF4 in stabilizing FNDC5 mRNA stability by generating a circ_AFF4/IGF2BP3/FNDC5 complex to promote osteogenesis (Supplementary Fig. 3). This finding may provide a novel theoretical basis for the application of circ_AFF4 in bone-formation-related diseases, such as osteoporosis.

## Electronic supplementary material

Below is the link to the electronic supplementary material.

Supplementary Material 1

Supplementary Material 2

## Data Availability

All data generated or analyzed during this study are included in this article. The datasets used and/or analyzed during the current study are available from the corresponding author on reasonable request.

## Abbreviations

FNDC5: fibronectin III domain-containing protein 5
PGC-1α: peroxisome proliferator-activated receptor γ coactivator 1α
circRNA: circulation RNA
KDM1A: lysine (K)-specific demethylase 1 A
IGF2BP3: insulin-like growth factor-2 mRNA-binding protein 3
RBP: mRNA-binding protein 3
BM-MSCs: bone marrow mesenchymal stem cells
DMEM: dulbecco´s modified Eagle´s Medium
RIP: immunoprecipitation
ALP: alkaline phosphatase
ARS: alizarin red S
FISH: fluorescence in situ hybridization
ELISA: enzyme-linked immunosorbent assay
EMSA: electrophoretic mobility shift assay
RT-qPCR: real-time polymerase chain reaction
IHC: immunohistochemistry
ChIP: chromatin immunoprecipitation
OM: osteogenic medium
NM: normal medium
