# Supplementary material for: CircRNA AFF4 induced by KDM1A promotes osteogenic differentiation through FNDC5/Irisin pathway
Source: Mol Med. 2022 Nov 18;28:134. doi: 10.1186/s10020-022-00557-7 (PMC9673395; doi:10.1186/s10020-022-00557-7)
Supplement: Supplementary file 1 — Supplementary Material 1 [file 10020_2022_557_MOESM1_ESM.docx]

Supplemental materials


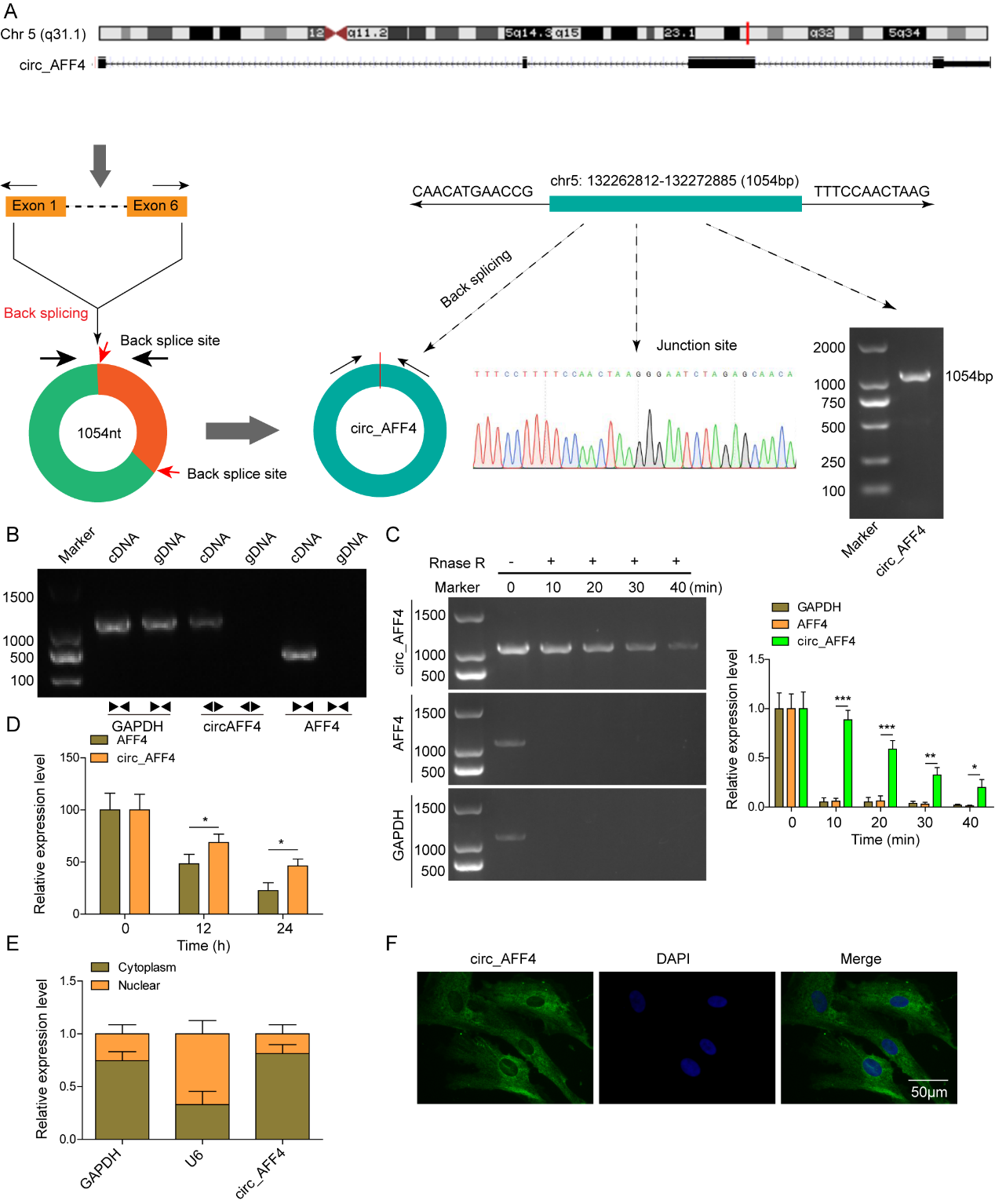


**Supplementary Figure 1 Characterization of circ_AFF4 in BM-MSCs**

(A) circ_AFF4 was derived from back-spliced exons 1-6 of AFF4. The back-splice junction of circ_AFF4 was identified by Sanger sequencing. (B)PCR analysis of circ_AFF4 and linear AFF4 in cDNA and gDNA. (C) RT-qPCR analysis of circ_AFF4 and linear AFF4 mRNA after RNase R treatment in BM-MSCs. (D) RT-qPCR analysis for the circ_AFF4 and AFF4 mRNA after Actinomycin D treatment in BM-MSCs after 0, 12 and 24 hours. (E) The expression of circ_AFF4 in the cytoplasm and nucleus of BM-MSCs was detected by cytoplasmic and nuclear fractionation experiments. (F) RNA fluorescence in situ hybridization for circ_AFF4. Cell nuclei were stained with DAPI. Scar bar = 50 µm. **p*<0.05, ***p*<0.001, and ****p*<0.001. Each experiment was performed at least three times independently.


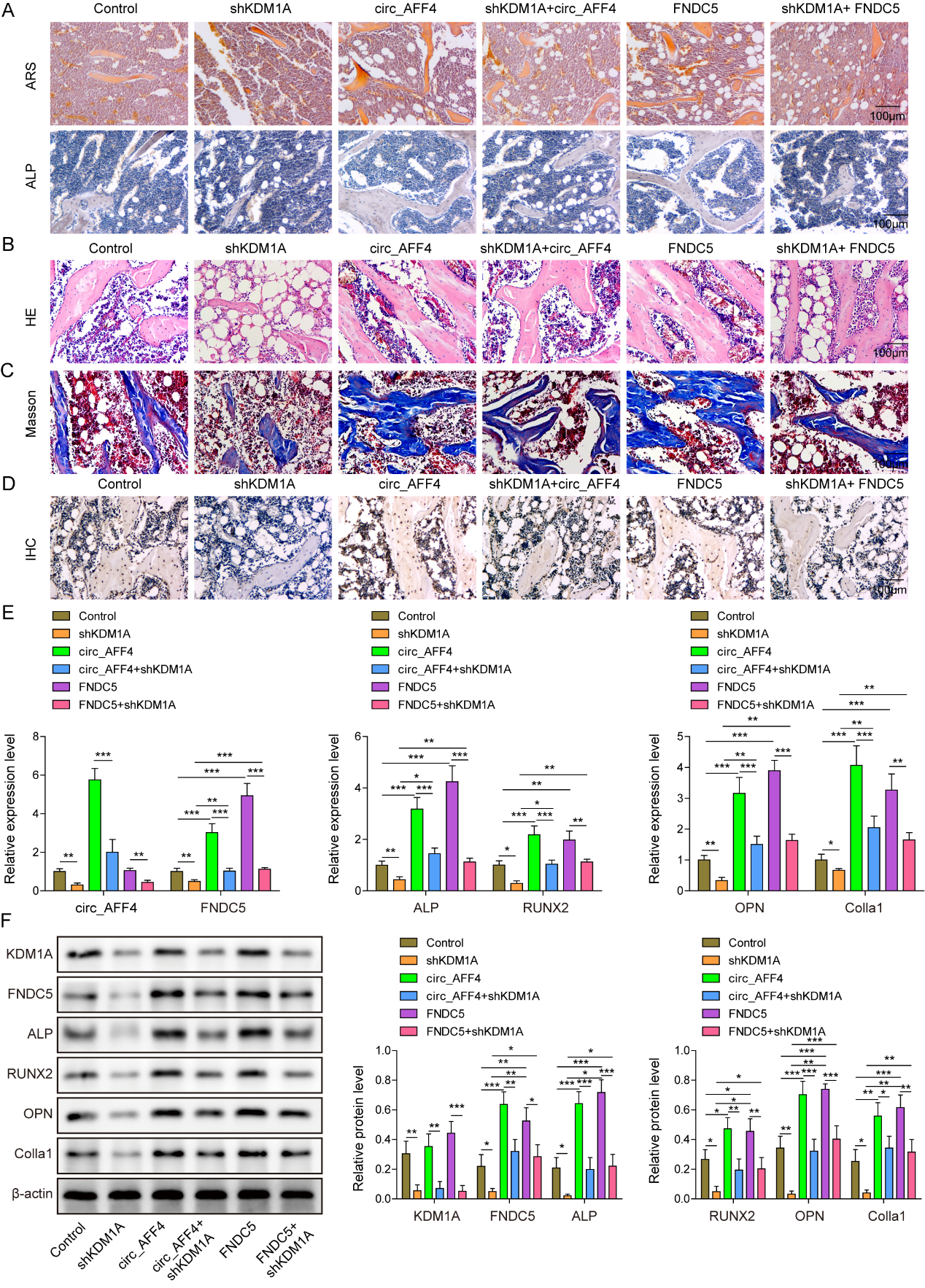


**Supplementary Figure 2 KDM1A enhances bone formation by regulating circ_AFF4 and FNDC5 *in vivo.***

BM-MSCs that transfected with sh-NC and lentiviruses (control), sh-KDM1A (shKDM1A, circ_AFF4 overexpression lentiviruses (circ_AFF4), sh-KDM1A plus circ_AFF4 overexpression lentiviruses (shKDM1A+circ_AFF4), FNDC5 overexpression lentiviruses (FNDC5), and sh-KDM1A plus FNDC5 overexpression lentiviruses (shKDM1A+circ_FNDC5) cultured in OM for 1 week. (A) ARS and ALP staining were performed to examine the bone cell formation in mice. Scar bar = 100 µm. (B) Representative H&E and (C) Masson staining images in mouse femurs showing the in vivo bone formation and collagen deposition. Scar bar = 100 µm. (D) Expression of the osteogenic differentiation-related transfection factor RUNX2 was evaluated using IHC staining. Scar bar = 100 µm. RT-qPCR (E) and Western blot (F) were used to investigate the osteogenic marker gene and protein expression. **p*<0.05, ** *p*<0.001, and ****p*<0.001. Each experiment was performed at least three times independently.


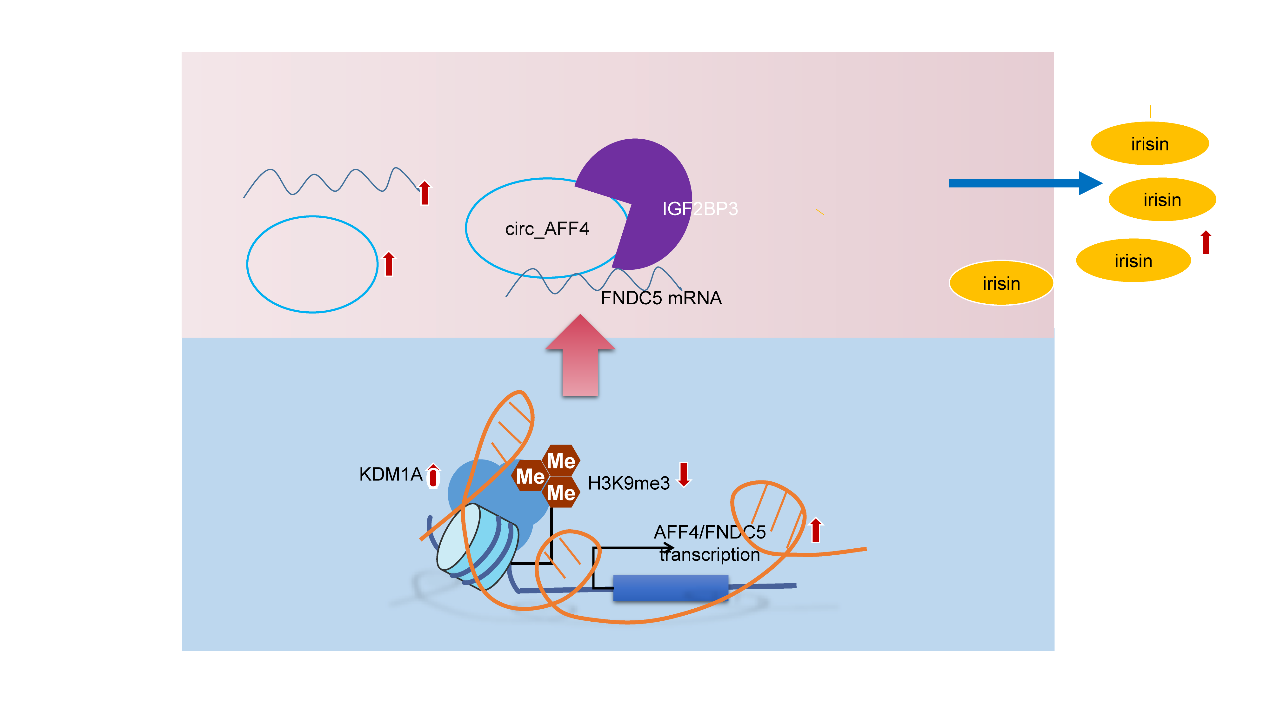


**Supplementary Figure 3. A model elaborating the critical role of circ_AFF4 in stabilizing FNDC5 mRNA stability by generating a circ_AFF4/IGF2BP3/FNDC5 complex to promote Irisin and osteogenesis.**
