# Supplementary material for: CircRNA AFF4 induced by KDM1A promotes osteogenic differentiation through FNDC5/Irisin pathway
Source: Mol Med. 2022 Nov 18;28:134. doi: 10.1186/s10020-022-00557-7 (PMC9673395; doi:10.1186/s10020-022-00557-7)
Supplement: Supplementary file 2 — Supplementary Material 2 [file 10020_2022_557_MOESM2_ESM.doc]

Reviewer reports:

Reviewer #2: The authors have responded to the questions and have significantly improved the manuscript.


Reviewer #3: The authors have extensively revised the manuscript in many places. Most of the points raised in the initial review were adequately satisfied. The Abstract and Methods sections are greatly improved and the Results section is much more clear. The revision of the Discussion is also much improved but the discussion of the limitations of this study is inadequate. It appears the authors have conflated the ideas of the questions that remain open in the field and what were the limitations of the current study. The text added on p. 24 (lines 34-40) ending in etc. should be revised. Moreover, a figure legend accompanying the model figure in Supplementary Figure 3 must be included.

**Reply:** Thanks for the comments. We have revised the limitations of this study. Please find the revised limitations in the second last paragraph. The etc. in the text was removed and the figure legend of supplementary figure 3 was updated based on the comments.
